# The Event-Specific Benefits of Writing About a Difficult Life Experience

**DOI:** 10.5964/ejop.2089

**Published:** 2021-02-26

**Authors:** Charles Matthew Stapleton, Hui Zhang, Jeffrey Scott Berman

**Affiliations:** a Department of Psychological Science, University of North Georgia, Oakwood, GA, USA; b Department of Psychology, Westfield State University, Westfield, MA, USA; c Department of Psychology, University of Memphis, Memphis, TN, USA; Trinity College Dublin, Dublin, Ireland

**Keywords:** expressive writing, specificity effects, cognitive avoidance, intrusive thoughts, positive emotions, negative emotions

## Abstract

Previous research demonstrates that writing about life’s difficult moments benefits the writer cognitively and emotionally. However, it is unclear whether the benefits of writing are specific to the event written about or whether the benefits are global. This study was designed to address this issue. Participants were 120 undergraduate students who had experienced at least two difficult life events. Participants were randomly assigned into experimental and control groups. Experimental participants wrote about one of these difficult events and control participants wrote about an interesting life event of their choosing. Experimental participants reported their positive and negative emotions as well as their cognitive avoidance and intrusion concerning the event written about and another event not written about. Control participants reported their emotions and cognitions concerning two difficult life events. All participants also reported their general distress. These assessments were done immediately after writing and one week later. The results indicated that experimental participants were emotionally stronger, less upset, and less cognitively avoidant about the particular difficult life event they wrote about compared to an event they did not write about. Similar comparisons between ratings of a written-about and a not-written-about event were not significant for passion, fear, and cognitive intrusion. There was evidence for a possible indirect effect of writing on general distress through changes in event-specific cognitions and emotions. Discussion of these results focuses on how writing may specifically help change a writer’s feelings and thoughts about a particular situation.

When facing difficult life experiences, such as natural disasters, health problems, violence, or emotional struggles, people find writing to be a beneficial tool to cope with difficulties. Individuals who write about difficult life experiences often report emotional and cognitive benefits from the writing (Frattaroli, 2006; Pavlacic, Buchanan, Maxwell, Hopke, & Schulenberg, 2019; Reinhold, Bürkner, & Holling, 2018). For instance, writing about difficulties improves long-term positive mood (Páez, Velasco, & Gonzalez, 1999) and supports overall psychological well-being (Park & Blumberg, 2002). Writing also reduces intrusion and avoidance symptoms (Klein & Boals, 2001; Radcliffe, Lumley, Kendall, Stevenson, & Beltran, 2007), posttraumatic stress symptoms (Hoyt & Yeater, 2011), self-criticism (Troop, Chilcot, Hutchings, & Varnaite, 2013), stress related to ambivalence (Kelly, Wood, Shearman, Phillips, & Mansell, 2012), rumination symptoms (Gortner, Rude, & Pennebaker, 2006), and depression and anxiety symptoms (Graf, Gaudiano, & Geller, 2008).

Just as therapists are trained to target a specific problem and evaluate progress, there is a practical need to evaluate how writing changes emotions and cognitions related to a particular experience. It is common that individuals are influenced by multiple difficult life experiences (Kessler, Sonnega, Bromet, Hughes, & Nelson, 1995). However, previous research has yet to address whether the emotional and cognitive benefits of writing are more salient for the specific experience written about than other experiences not written about, or whether the benefits apply broadly to all difficult events the writer may have experienced. The purpose of the current study is to evaluate the specific cognitive and emotional benefits of writing about a difficult life experience. In the following section, we review (1) a brief history of the expressive writing paradigm, (2) the psychological benefits of expressive writing, (3) some possible mechanisms by which writing benefits the writer, and (4) three issues related to specificity in expressive writing that we address in the present study.

## Expressive Writing

Expressive writing refers to the procedure of writing freely to express emotions about an experience without focusing on grammar or spelling (Reinhold et al., 2018). In one of the first systematic expressive writing studies, Pennebaker and Beall (1986) randomly assigned undergraduate students to write about either a difficult life event or another neutral topic. Those asked to write about a difficult event had significantly fewer medical visits at a 6-month follow-up compared to those asked to write about a neutral event. Pennebaker and Beall (1986) presented evidence showing that writing about a difficult event affects the writer differently than writing about a neutral topic.

Many studies evaluating the effect of writing about life’s difficulties have used an experimental design like the one used by Pennebaker and Beall (1986). Typically, this design involves having participants in the experimental group write about a difficult, potentially traumatic life experience and those in the control group write about a neutral topic on 3–4 consecutive days for 15–20 min per session. Expressive writing has been used widely as a therapeutic intervention by educational, counseling, and clinical psychologists.

## Psychological Benefits of Expressive Writing

Previous research has supported that expressive writing is a beneficial tool for writers’ mental and physical health. While consistent benefits of expressive writing on individuals’ physical health outcomes (e.g., illness-related visits to doctor) have been found in previous research, the psychological benefits of writing are not as consistent (Baikie & Wilhelm, 2005). Overall, meta-analysis reviews have indicated a small to medium effect of expressive writing on psychological outcomes (Frattaroli, 2006; Pavlacic et al., 2019).

Some researchers have found immediate cognitive and emotional changes after writing. For example, some have documented an increase in negative mood and a decrease in positive mood immediately after writing about a life experience (Baikie & Wilhelm, 2005). Some researchers also found long-term cognitive and emotional changes after writing about difficult life experiences. For example, individuals may experience more positive mood (Páez et al., 1999; Pennebaker, Kiecolt-Glaser, & Glaser, 1988), improved psychological well-being (Park & Blumberg, 2002), less intrusive and avoidant thoughts (Klein & Boals, 2001), and less depressive symptoms (Lepore, 1997). However, not all researchers have found these changes (Deters & Range, 2003; Meads & Nouwen, 2005; Mogk, Otte, Reinhold-Hurley, & Kröner-Herwig, 2006; Stroebe, Stroebe, Schut, Zech, & van den Bout, 2002).

## Mechanisms of Expressive Writing’s Benefits

Several mechanisms may be responsible for the benefits of writing about life’s ups and downs. Writing about life’s problems allows for the disclosure of the secret and often hurtful places in our lives. Early on, Pennebaker and Beall (1986) speculated that *emotional catharsis* could explain many of the observed effects. Later, Pennebaker (1993) argued that writing about a difficult life experience helps the writer let go of thoughts and feelings. Actively inhibiting thoughts and feelings concerning a distressful event puts strain on the body. Writing about a difficult event can help reduce the mental and physical workload of inhibition. This process is called *disinhibition*. More recently, it has been suggested that writing about a trauma is like *exposure* therapy (Sloan & Marx, 2006; Sloan, Marx, & Epstein, 2005). After exposure to negative feelings and thoughts repeatedly, expressive writing may lead to habituation and produce extinction of these negative feelings and thoughts (Lepore, Greenberg, Bruno, & Smyth, 2002; Rankin et al., 2009).

Writing about a difficult event may encourage individuals to use more *cognitive processing* words (e.g., understand, realize, know, reason). The use of these words may encourage deeper cognitive processing of life’s struggles (Ullrich & Lutgendorf, 2002). Writing may help individuals make sense of the upsetting event, organize their experiences, and develop a coherent narrative concerning the event (Graybeal, Sexton, & Pennebaker, 2002; Pennebaker & Seagal, 1999; Smyth, True, & Souto, 2001). Through the writing process, individuals integrate these experiences into their self-schema and develop a more positive sense of self (Harber, Pennebaker, & Christianson, 1992).

Writing strengthens *self-regulation* processes and promotes control and mastery of distressful thoughts and feelings (King, 2002; Lepore et al., 2002; Nückles, Hübner, & Renkl, 2009). Self-regulated learning theories have their roots in both social-cognitive theory and socio-cultural theory (Schunk & Zimmerman, 2012; Zimmerman, 1989). Self-regulated learning involves learning by watching and interacting with others. Initially, because every learning situation is new, a novice learner is monitored, observed, and given direction by a more experienced learner. The novice learner becomes a skilled learner by repeating what they observe and completing the instructions given. When the learner sees themself completing tasks successfully several times their self-efficacy grows for the task. The learner understands how knowledge used in one task may be applied to other similar tasks. Feelings of pride and strength are associated with the learner’s growing self-efficacy (Pekrun, Goetz, Titz, & Perry, 2002). The learner must learn how to regulate feelings of distress and being upset that accompany the process of learning and must learn how to directly solve problems rather than avoid them.

Writing can be the mechanism through which the self-regulated learning process transpires (Graham, Harris, MacArthur, & Santangelo, 2018; Schmitz, Klug, & Schmidt, 2011). Writing allows individuals to observe, monitor, and evaluate how they express and control their emotions. The sense of control over emotions that is a direct result of writing helps the writer improve their well-being and reduces negative emotions. From the self-regulated learning perspective, a writer would first gain mastery over emotions and thoughts associated with the life event that is written about. At a later point, when the writer has learned how to apply the skills to other events, there would be improvement in overall functioning.

All the theories about expressive writing partially account for the psychological changes observed after writing. There is evidence in support of and against each mechanism (Frattaroli, 2006). Many of the theories are compatible with one another. The most empirical support has been found for the exposure, disinhibition, and self-regulation perspectives. However, emotional catharsis and cognitive-processing have the least support of the mechanisms mentioned (Frattaroli, 2006).

## Specificity in Expressive Writing

There is a need to address three issues related to the specificity regarding the benefits of expressive writing. First, the benefits of writing may be associated with specific life experiences. In the expressive writing research, participants are asked to write about specific events. However, it is common for individuals to experience multiple difficult life experiences (Kessler et al., 1995). Yet, research has not explored how writing about a specific event benefits the lives of individuals who have experienced multiple difficult experiences. It might be the case that writing about a life challenge modifies thoughts and feelings concerning only the event written about. For example, Fivush, Edwards, and Mennuti‐Washburn (2003) asked participants to write about their emotional reactions to 9/11 within two months of the event, and then write about 9/11 again one month later, and again six months later. Participants who used more cognitive-processing and emotional words in their writing reported coping better with 9/11 than others who used fewer cognitive and emotional words in their writing. Writing about 9/11 specifically helped the writers cope with their feelings concerning that event (Fivush et al., 2003).

It might also be the case that writing about a difficult event produces a global improvement for both the event written about and other events not written about. For example, Pavlacic et al. (2019)’s meta-analysis suggested that writing improves participants’ overall quality of life. It is likely that there are initial changes in the cognitions and emotions about a particular life event, and then these changes are applied to other similar experiences. The change that occurs when writing about a particular event might be transferred to the skills a person uses to deal with other events. The self-regulation perspective can clarify this transference. Individuals learn how to effectively cope with one event, and then apply their new abilities to similar situations. However, little research has examined both the specific and global psychological effects of writing.

Second, we should consider the specificity of the emotions and thoughts changed by writing. Our emotions and cognitions are contextual and directed toward particular people, events, and objects. This has been called the intentional structure (i.e., *aboutness*) of emotions and cognitions (Denzin, 1984; Ratcliffe, 2008). For instance, a person who is upset might be upset *about* the car accident they were in last week or *about* their father’s passing after having cancer.

Related to their intentional structure, emotions can be classified both broadly and narrowly according to the types of social problems they help solve (Keltner & Gross, 1999). For instance, positive and negative emotions are differently associated with our attention, memory, and behavioral tendencies (Fredrickson, 1998; Fredrickson & Branigan, 2005). This distinction between positive and negative emotions is broad and based on the valence of emotions (i.e., pleasant or unpleasant).

However, emotions can be classified in other ways according to more narrow functions (Egloff, Schmukle, Burns, Kohlmann, & Hock, 2003; Mihić, Novović, Čolović, & Smederevac, 2014). Some emotions are closely tied to a person’s self-concept (Götz, Cronjäger, Frenzel, Lüdtke, & Hall, 2010). For example, if a person feels strong, proud, or determined about themself, they will feel positive and ready to endure whatever life brings (Williams & DeSteno, 2008). Other positive emotions are closely tied to the interest, excitement, and passion invested in activities (Götz et al., 2010). For example, a person who feels passionate will seek out opportunities to reengage with the experiences, people, and objects that they are passionate about. Although emotions related to the strength of one’s self-concept and emotions conveying passion are both positive emotions, they serve different social functions. Someone might feel strong after undergoing a difficult experience and productively processing that experience. But it is unlikely that someone would feel passionate or excited about a difficult life experience no matter how well they have dealt with it. We expected that writing would influence event-specific positive emotions related to strength, but not passion and being energetic.

Similarly, there are different facets of negative emotions with distinct social functions (Mehrabian, 1997). Some negative emotions, including being upset or angry, draw our attention to losses such as the loss of a loved one or a desired opportunity. Other negative emotions, such as fear, draw our attention to threats. Previous empirical studies suggest two facets of negative emotions—upset and fear (Allan, Lonigan, & Phillips, 2015; Eadeh, Breaux, Langberg, Nikolas, & Becker, 2020; Killgore, 2000). Because many people choose to write about losses instead of threats, we expected that our writing intervention would reduce negative emotions related to being upset about a past situation, but not reduce fear.

Cognitions also have an intentional pattern (i.e., *aboutness*). For example, a man avoids thinking *about* the fight he had with his spouse last week, or a woman has intrusive thoughts *about* an abusive relationship. Since the previous literature suggests that writing generally reduces cognitive avoidance and intrusive thoughts (Klein & Boals, 2001; Radcliffe et al., 2007), we expected that writing would reduce perceptions of cognitive avoidance and intrusion concerning the events written about in comparison to other events not written about. Many studies (e.g., Baikie & Wilhelm, 2005; Reinhold et al., 2018) have looked at how writing changes less contextualized moods and thoughts instead of the specific emotions and cognitions about a difficult life event. This study is one of the first to examine how writing changes the emotions and cognitions connected to a specific event.

Third, we should consider the specificity of the writing context. In expressive writing research, participants are often asked to write about a difficult life event for 3–5 sessions, for 15–20 minutes in a laboratory (Pennebaker & Beall, 1986). This is a controlled setting that improves the internal validity of the writing intervention. In contrast to this controlled setting, students and clients may be asked to write about difficult life experiences in their home to reduce depression and anxiety (Collins & Dozois, 2008). Writing is also commonly prescribed by self-help books. As such, writing may be used daily or intermittently. As authors, it is our experience that we use journaling about both positive and negative life experiences intermittently to focus on events that punctuate our lives rather than as an on-going activity. Additionally, we have observed that clients and students write about their problems irregularly even when assigned to do so daily. Although the therapeutic effect of writing may be maximized by having participants write on multiple occasions and for lengthy periods, it is also important to know the effect of short, intermittent writing episodes. Previous research has investigated short writing interventions, but more work needs to be done (Burton & King, 2008). Short and intermittent writing episodes are closer to how writing will be used in writers’ real-life experiences and this is related to the external validity of the intervention. Further evaluation of short writing interventions may provide implications for practitioners to monitor students’ and clients’ progress.

## The Present Study

Based on the literature and the previous three considerations concerning specificity, we hypothesized that:

1. Immediately after writing, those who wrote about a difficult life experience would feel (a) more strength-related positive emotions and (b) less upsetting negative emotions about the experience they wrote about in comparison to another difficult experience they did not write about.
2. Immediately after writing, those who wrote about a difficult life experience would report (a) less cognitive avoidance and (b) less cognitive intrusion concerning the experience they wrote about in comparison to another difficult experience they did not write about.
3. Given the brevity of our writing intervention, we did not expect that the writing topic (a difficult life event vs. an interesting life event) would have a direct impact on general distress either immediately after writing or one week later.
4. However, we do believe that writing about a difficult experience influences the writer’s general distress indirectly. Based on the self-regulated learning perspective, we propose that writing about a difficulty leads first to changes in the cognitions and emotions related to that particular difficulty, and then those specific changes are transferred to other similar situations producing a global change in distress. Thus, we were interested in exploring whether the event-specific emotions and cognitions immediately after writing were associated longitudinally with general distress reported one week later.

To address these hypotheses, we asked participants to write about either a difficult life experience or an interesting life experience. Experimental participants wrote about a difficult life experience and reported their general distress in addition to cognitions and emotions for both the event written about and another difficult event not written about. Control participants wrote about an interesting event and reported their general distress in addition to cognitions and emotions for two difficult life events. For all groups of participants, cognitive and emotional changes as well as general distress were assessed again one week later.

## Method

### Participants

The participants were 120 college students (65% female) recruited from undergraduate psychology courses at a large urban university in the United States. The mean age of participants was 20.84 years (*SD* = 4.78, range = 17–50). Reported ethnicity was 48% European American, 43% African American, 4% Latinx American, 1% Asian American, and 4% other ethnicities. The study was approved by the Institutional Review Board of the university.

### Procedure

Although the procedures of the study are fully described here, readers should consult the online supplement for ancillary material and information. At the beginning of two academic terms, students were administered a prescreening questionnaire. To maintain confidentiality, we divided this questionnaire into two parts. The first part asked students to provide their names and contact information. The second asked students to list three difficult or emotionally painful life events they had gone through or were currently going through. Individuals were also asked to indicate how much distress each event was currently causing them using a 10-point scale with verbal labels of *least distressing* (1) and *most distressing* (10). Only individuals who could list at least two unrelated events of 4 or greater on the distress scale were eligible to participate in the study.

Students who met the selection criteria were contacted and given the opportunity to take part in the study. When participants reported to the lab, they were told as part of the informed consent procedure that participation in the study would require them to write about their life experience. Identifying information was collected separately from the writing samples, making the identity of any writing sample confidential.

Before coming to the lab, each participant was randomly assigned to one of two experimental groups or a control group. Participants in the first experimental group (*n* = 41) wrote about the most distressing experience identified by the participant on the prescreening questionnaire. Participants in the second experimental group (*n* = 39) wrote about the second most distressing experience from the prescreening questionnaire. Participants in the control group (*n* = 40) wrote about an interesting experience of their choosing.

Individuals participated in the study one at a time. After giving consent, the participant was presented with the writing instructions (see Supplementary Materials). Each participant was given 10-min to write, after which there was a 10-min break. After the break, the participant continued writing for another 10-min. After writing, all participants were asked to indicate their cognitions for the most and second-most difficult life experiences identified on the prescreening questionnaire using two forms of the Impact of Events Scale (IES; Horowitz, Wilner, & Alvarez, 1979), a questionnaire designed to measure participants’ perceptions of the avoidance and intrusion associated with a particular event. Then, all participants indicated their emotions for the most and second-most difficult life experiences identified on the screening questionnaire using two forms of the Positive and Negative Affect Schedule (PANAS; Watson, Clark, & Tellegen, 1988), a brief measure designed to assess the emotions of an individual. The sequence of the two forms of the IES and PANAS was counterbalanced such that half of the participants first reported on the most distressing event and half of the participants the second-most distressing event. All participants also completed the Hopkins Symptom Checklist-25 (HSCL-25; Hesbacher, Rickels, Morris, Newman, & Rosenfeld, 1980), a self-report measure designed to assess general distress. When the participants returned to the lab one week later, they completed all the questionnaires from the previous appointment. All 120 participants returned to complete the follow-up questionnaires.

### Measures

#### Impact of Events Scale

The IES (Horowitz et al., 1979) is a self-report questionnaire with 15 items designed to measure cognitions related to a particular event. The IES can be used to ask the respondent to answer the items regarding a particular life event. Respondents indicated how often they reacted to the distressing situation in 15 ways in the last seven days. Participants in this study used the original 4-point rating scale, recommended by Horowitz et al. (1979), that includes the values 0 (*not at all*), 1 (*rarely*), 3 (*sometimes*), and 5 (*often*). We retained the original discontinuous rating scale so comparisons with previous research are possible. The avoidance subscale includes 8 items and measures attempts to not think about the event. The intrusion subscale includes 7 items and measures unwelcome involuntary thoughts or images about the event. Cronbach’s alphas for the avoidance subscale ranged from .77 to .83, and for the intrusion subscale they ranged from .85 to .87, across the two assessment points and two experiences in this study.

#### Positive Affect and Negative Affect Schedule

The PANAS (Watson et al., 1988) is a 20-item measure consisting of 10 items measuring positive emotions and 10 items measuring negative emotions. Each item of the measure consists of a single descriptor of an emotion. Respondents indicated the degree to which they felt each of the emotions at the moment in reference to the specified event, using a 5-point scale, including the values 1 (*very slightly or not at all*), 2 (*a little*), 3 (*moderately*), 4 (*quite a bit*), and 5 (*extremely*).

As researchers we are more interested in discrete subgroups of positive and negative emotions (e.g., emotions related to threat, strength of self-concept, loss, and activity) than broad classifications of emotions (e.g., positive and negative emotions). Therefore, during the analysis phase of this study, it was decided that our presentation of the results would reflect this interest. It has been suggested that the positive affect subscale may be divided into separate facets (Egloff et al., 2003; Mihić et al., 2014). In this study, the positive affect items were divided into a *strength* subscale (strong, determined, proud, inspired, and active) and a *passion* subscale (excited, interested, attentive, enthusiastic, and alert). Cronbach’s alphas for the *strength* subscale ranged from .82 to .86, and for the *passion* subscale they ranged from .78 to .82 in this study. The negative affect items may be further divided into an *upset* subscale (distressed, irritable, hostile, and upset) and a *fear* subscale (scared, nervous, afraid, guilty, ashamed, and jittery) (Allan et al., 2015; Eadeh et al., 2020; Killgore, 2000; Mehrabian, 1997). Cronbach’s alphas for the *upset* subscale ranged from .78 to .82, and for the *fear* subscale they ranged from .74 to .83 in this study.

#### Hopkins Symptom Checklist-25

The HSCL-25 (Hesbacher et al., 1980) is a 25-item self-report measure of general distress which includes psychological and somatic indicators of distress. On this measure, respondents report symptom severity during the past week on a 4-point scale, including 1 (*not at all*), 2 (*a little*), 3 (*quite a bit*), and 4 (*extremely*). For this study, Cronbach’s alpha immediately following writing was .91 and one week later was .92.

### Data Analysis

The analysis proceeded in two steps. The purpose of the first step was to understand the event-specific effects of writing about a difficult life experience. In the first step, the focus was on only those who wrote about a difficult life event (*n* = 80). This analysis compared scores for the event experimental participants wrote about with the scores for the event they did not write about. This was a within-subjects comparison.

The purpose of the second step was to assess the direct effect of writing on general distress and to probe for the indirect effect of writing on general distress through the event-specific emotions and cognitions. In this step, those who wrote about a distressing experience (*n* = 80) were compared to those who wrote about an interesting event (*n* = 40) on a measure of general distress. This provided an assessment of the direct effect of writing topic on general distress. Then, we explored the possibility that thoughts and feelings concerning the written-about experience may be associated with general distress. This was meant to provide supporting evidence for a possible indirect effect of writing on general distress through changes in event-specific cognitions and emotions.

## Results

The issue addressed by the first step of the analysis was whether the effect of writing about a difficult life event was specific to the event written about. To test our hypotheses (1a-b) and (2a-b), we conducted separate 2 (Time: Immediate vs. 1-Week) × 2 (Event: Written-About vs. Not-Written-About) Repeated Measures Analyses of Variance for the strength, passion, upset, and fear emotion subscales and for the avoidance and intrusion cognitive subscales. The results of the analyses for the positive emotions are contained in Table 1, the results for the negative emotions in Table 2, and the results for the cognitions in Table 3.

**Table 1:** Means, Standard Errors (in Parentheses) and ANOVA Results for Positive Emotions (Strength and Passion Subscales)

Time (T)	Event (E)			ANOVAs		
	Written	Not-written	Total	Effect	*F*(1, 79)	$\eta_{p}^{2}$
Strength						
Immediate	${\mathrm{2.84}}_{a}^{d}$ (0.13)	${\mathrm{2.53}}_{b}^{f}$ (0.12)	2.69 (0.11)			
1-Week	${\mathrm{2.47}}_{c}^{e}$ (0.13)	${\mathrm{2.43}}_{c}^{f}$ (0.13)	2.45 (0.11)			
Total	2.66 (0.12)	2.48 (0.12)				
				T	9.54**	.11
				E	2.56	.03
				T × E	6.19*	.07
Passion						
Immediate	${\mathrm{2.60}}_{a}^{d}$ (0.12)	${\mathrm{2.52}}_{a}^{f}$ (0.11)	2.56 (0.10)			
1-Week	${\mathrm{2.36}}_{c}^{e}$ (0.12)	${\mathrm{2.34}}_{c}^{g}$ (0.11)	2.35 (0.10)			
Total	2.48 (0.11)	2.43 (0.10)				
				T	12.49***	.14
				E	0.20	.01
				T × E	0.57	.01

*Note*. *n* = 80. Subscripts and superscripts reflect the results of follow-up comparisons. Subscripts reflect the row-wise written vs. not-written comparisons. Means compared within rows that do not share an identical subscript differ significantly. Superscripts reflect column-wise immediate vs. 1-week comparisons. Means compared within columns that do not share an identical superscript differ significantly. ANOVA = analysis of variance.

*p < .05. **p < .01. ***p < .001.

**Table 2:** Means, Standard Errors (in Parentheses) and ANOVA Results for Negative Emotions (Upset and Fear Subscales)

Time (T)	Event (E)			ANOVAs		
	Written	Not-written	Total	Effect	*F*(1, 79)	$\eta_{p}^{2}$
Upset						
Immediate	${\mathrm{2.57}}_{a}^{d}$ (0.11)	${\mathrm{2.80}}_{b}^{f}$ (0.12)	2.69 (0.10)			
1-Week	${\mathrm{2.32}}_{c}^{d}$ (0.13)	${\mathrm{2.32}}_{c}^{g}$ (0.13)	2.32 (0.10)			
Total	2.45 (0.09)	2.56 (0.10)				
				T	11.71***	.13
				E	1.44	.02
				T × E	4.30*	.05
Fear						
Immediate	${\mathrm{2.16}}_{a}^{d}$ (0.10)	${\mathrm{2.17}}_{a}^{f}$ (0.09)	2.17 (0.08)			
1-Week	${\mathrm{1.95}}_{c}^{e}$ (0.09)	${\mathrm{2.00}}_{c}^{g}$ (0.10)	1.98 (0.08)			
Total	2.06 (0.09)	2.09 (0.09)				
				T	7.32**	.09
				E	0.10	.01
				T × E	0.10	.01

*Note*. *n* = 80. Subscripts and superscripts reflect the results of follow-up comparisons. Subscripts reflect the row-wise written vs. not-written comparisons. Means compared within rows that do not share an identical subscript differ significantly. Superscripts reflect column-wise immediate vs. 1-week comparisons. Means compared within columns that do not share an identical superscript differ significantly. ANOVA = analysis of variance.

*p < .05. **p < .01. ***p < .001.

**Table 3:** Means, Standard Errors (in Parentheses) and ANOVA Results for Cognitions (Avoidance and Intrusion Subscales)

Time (T)	Event (E)			ANOVAs		
	Written	Not-Written	Total	Effect	*F*(1, 79)	$\eta_{p}^{2}$
Avoidance						
Immediate	${\mathrm{2.19}}_{a}^{d}$ (0.08)	${\mathrm{2.44}}_{b}^{f}$ (0.08)	2.32 (0.06)			
1-Week	${\mathrm{2.08}}_{c}^{e}$ (0.07)	${\mathrm{2.18}}_{c}^{g}$ (0.08)	2.13 (0.07)			
Total	2.13 (0.07)	2.31 (0.08)				
				T	17.57***	.18
				E	5.25*	.06
				T × E	5.12*	.06
Intrusion						
Immediate	${\mathrm{2.63}}_{a}^{d}$ (0.09)	${\mathrm{2.69}}_{a}^{f}$ (0.09)	2.66 (0.07)			
1-Week	${\mathrm{2.34}}_{c}^{e}$ (0.09)	${\mathrm{2.33}}_{c}^{g}$ (0.08)	2.34 (0.07)			
Total	2.49 (0.08)	2.51 (0.08)				
				T	28.67***	.27
				E	0.11	.01
				T × E	0.60	.01

*Note*. *n* = 80. Subscripts and superscripts reflect the results of follow-up comparisons. Subscripts reflect the row-wise written vs. not-written comparisons. Means compared within rows that do not share an identical subscript differ significantly. Superscripts reflect column-wise immediate vs. 1-week comparisons. Means compared within columns that do not share an identical superscript differ significantly. ANOVA = analysis of variance.

*p < .05. **p < .01. ***p < .001.

### Positive Emotions

#### Strength

For the strength subscale, there was a significant Time × Event interaction (see Table 1, upper panel). Immediately after writing, the writers reported feeling more strength about the event they wrote about compared to the event they did not write about. However, this specificity effect was not evident one week later. The main effect for Time was also significant, but the main effect for Event was not.

#### Passion

For the passion subscale, the main effect for Time was significant (see Table 1, lower panel). This indicated that ratings of passion went down from immediately after writing to one week later. However, neither the main effect for Event, nor the Time × Event interaction were significant.

Our Hypothesis (1a) was supported for the positive emotions. Experimental participants reported more intense positive emotions for an event that they wrote about than an event that they did not write about. This was only true for positive emotions related to strength but not for positive emotions related to passion. The specificity effect for positive emotions related to strength was only found immediately after writing but not one week later.

### Negative Emotions

#### Upset

For the upset subscale, there was a significant Time × Event interaction (Table 2, upper panel). Immediately after writing, the writers reported feeling less upset about the event they wrote about compared to the event they did not write about. However, this specificity effect was not evident one week later. The main effect for Time was also significant, but the main effect for Event was not.

#### Fear

For the fear subscale, the main effect for Time was significant (Table 2, lower panel). This indicated that ratings of fear went down from immediately after writing to one week later. However, neither the main effect for Event, nor the Time × Event interaction effect was significant.

Our Hypothesis (1b) concerning negative emotions was supported. Experimental participants reported less intense negative emotions for an event that they wrote about than an event that they did not write about. This was only true for negative emotions related to being upset and not for negative emotions related to fear. The specificity effect for negative emotions related to being upset was only found immediately after writing and not one week later.

### Cognitions

#### Avoidance

For the cognitive avoidance subscale, there was a significant Time × Event interaction (Table 3, upper panel). Immediately after writing, the writers reported less avoidance for the event that they wrote about than the event that they did not write about. However, this specificity effect was not evident one week later. The main effect for Time, and the main effect for Event were also significant.

#### Intrusion

For the cognitive intrusion subscale, the main effect for Time was significant (Table 3, lower panel). This indicated that ratings of intrusive thoughts went down from immediately after writing to one week later. However, neither the main effect for Event, nor the Time × Event interaction effect was significant.

Our Hypothesis (2) concerning event-specific cognitions was partially supported. Consistent with our Hypothesis (2a), experimental participants reported avoiding their thoughts less for an event that they wrote about than an event that they did not write about. However, inconsistent with our Hypothesis (2b), there were no significant differences between ratings of intrusive thoughts for an event written about versus ratings of an event not written about.

### General Distress

To test our Hypothesis (3), the second step of the analysis compared the effect of writing topic on participants’ general distress. To do this, we conducted a 2 (Writing Topic: Difficult Experience vs. Interesting Experience) × 2 (Time: Immediate vs. 1-Week) Mixed Analysis of Variance on the ratings of the HSCL-25. There was a significant main effect for Time, *F*(1, 118) = 25.57, *p* < .001, $\eta_{p}^{2}$ = .10. This indicated that ratings of distress decreased from immediately after writing (*M* = 2.31, *SE* = 0.07) to one week later (*M* = 2.10, *SE* = 0.07). However, neither the Writing Topic main effect, *F*(1, 118) = 0.24, *p =* .62, $\eta_{p}^{2}$ < .01, nor the Writing Topic × Time interaction effect, *F*(1, 118) = 0.50, *p =* .48, $\eta_{p}^{2}$ = .01, was significant.

We also investigated whether ratings of emotions and cognitions made immediately after writing were associated with general distress one week later (Hypothesis 4). In this analysis, we focused on the experimental participants. To do this, we calculated correlations between emotions and cognitions immediately after writing and the 1-week general distress score (HSCL-25). We used Steiger’s (1980) method to compare two dependent associations: (1) the association of participants’ general distress with their immediate thoughts and feelings concerning the event written about; and (2) the association of participants’ general distress with their immediate thoughts and feelings concerning an event they did not write about. This method assesses whether the correlations of two predictors with a criterion variable differ significantly (*r*_jk_ versus *r*_jh_), while considering the weight of the correlation between the two predictors (*r*_kh_). In this study, the two predictors were the ratings made for an event written about and ratings made for an event not written about. The criterion variable was general distress one week later. The results of these analyses are presented in Table 4. As can be seen, negative emotions and cognitions related to the event discussed in the writing were significantly and positively correlated with general distress. Significant correlations between thoughts and feelings concerning an event not written about and general distress were less common.

**Table 4:** Comparisons of Correlations Between Emotions and Cognitions Made Immediately After Writing and Ratings of General Distress (HSCL-25) Made One Week Later

Subscale	Written T1 with Not Written T1 (*r*_kh_)	Written T1 with HSCL T2 (*r*_jk_)	Not Written T1 with HSCL T2 (*r*_jh_)	*z*-score (*r*_jk_ vs. *r*_jh_)
Positive emotions				
Strength	.534**	.002	-.016	0.16
Passion	.436***	.071	.080	-0.08
Negative emotions				
Upset	.494***	.327**	.110	1.97*
Fear	.409***	.239*	.238*	0.01
Cognitions				
Avoidance	.295**	.222*	.091	0.99
Intrusion	.259*	.355***	.221*	1.03

*Note. n* = 80. HSCL = 25-item version of Hopkins Symptom Checklist; T1 = immediate ratings; T2 = 1-week ratings.

*p < .05. **p < .01. ***p < .001.

Examining the comparisons between the written-about and not-written-about conditions, the *z*-score for being upset was significant. Ratings of being upset for the event written about was positively and significantly associated with general distress one week later; however, this correlation was not significant for the event that was not written about. These two correlations were significantly different.

## Discussion

Writing is a powerful tool for helping people heal from difficult life experiences (Baikie & Wilhelm, 2005). Usually the global effect of expressive writing on psychological outcomes has been evaluated (Park & Blumberg, 2002; Pavlacic et al., 2019). Because individuals commonly experience multiple difficult life events (Kessler et al., 1995), it is worthwhile to evaluate whether writing helps them cope with a specific event compared to other life difficulties. The findings of this study highlight the specific benefits writing has on the event written about and provides implications for theory, research, and practice.

### Event-Specific Benefits of Writing

Most importantly, the results of this study showed that participants in the experimental groups felt stronger, less upset, and less avoidant of the difficult life event that they just wrote about compared to another difficult life event they did not write about. First, the most significant result relates to strength immediately after writing. Based on research concerning the social functions of emotions (Keltner & Gross, 1999), we distinguished two subtypes of positive emotions: strength and passion. Although both are pleasant emotions, they can be distinguished by the adaptive functions they serve (Götz et al., 2010; Williams & DeSteno, 2008). Whereas strength is associated with being proud of oneself, being determined to complete tasks, and being inspired; passion is associated with being interested, excited, and enthusiastic about an activity. The results support our Hypothesis (1a): Participants in the experimental group reported more emotional strength associated with the event written about. However, they did not report feeling more passionate about the event they wrote about than another difficulty they did not write about. This suggests that writing’s specific initial benefits are associated with emotions related to self-concept more than activity-related emotions. Writers feel stronger, more determined, and prouder of themselves, not necessarily more ready or excited to act. This helps writers perform tasks in an ordered manner and persist through difficulties (Szagun & Schauble, 1997; Van Osch, Zeelenberg, & Breugelmans, 2018). The finding is consistent with the self-regulation hypothesis concerning the mechanism through which writing produces its benefits (King, 2002; Lepore et al., 2002). Writing about a difficult event allows writers to observe, express, and control their emotions associated with the difficult event and develop a sense of self-efficacy.

Similarly, following previous research, we distinguished two facets of negative emotions: upset and fear (Allan et al., 2015; Eadeh et al., 2020; Killgore, 2000). The results support our Hypothesis (1b): Participants in the experimental group reported being less upset regarding the event they wrote about compared to a difficult event they did not write about. However, they did not report feeling less fearful. One possible explanation is that the writing task did not address the social functions associated with fear. Fear helps provide an alarm to threats (Frijda, 2013). But many of our writers wrote about losses rather than threats to their survival and security. If we had asked our writers to write about their most frightening or threatening experiences, then we might have found event-specific effects for fear. In contrast, being upset is associated with feeling irritated, distressed, or hostile when the event is mentioned. This is consistent with the disinhibition hypothesis about writing’s benefits (Lepore & Smyth, 2002). Although culture, workplace values, and family mores may teach us to inhibit our feelings, inhibition of thoughts and feelings regarding an upsetting event takes effort and can be harmful. Writing helps participants express these inhibited feelings in a safe environment and in this way reduces stress. The increase in event-specific feelings of strength and the decrease in event-specific feelings of being upset may indicate an overall sense of emotional stability. This means that brief writing interventions may effectively change event-specific emotions promoting emotional stability regarding a specific event.

Consistent with our Hypothesis (2a), immediately after writing, participants in the experimental group reported fewer avoidant thoughts regarding the experience they wrote about. However, our Hypothesis (2b) was not supported: Participants in the experimental group did not report any difference in intrusive thoughts between the event written about and the event not written about. Previous studies have found that writing reduces both intrusion and avoidance symptoms (Klein & Boals, 2001; Radcliffe et al., 2007). These findings are also consistent with the disinhibition hypothesis. Intrusive and avoidant thoughts are signs of unresolved stress and that one is engaging in ongoing emotional inhibition. Writing provides an opportunity for writers to disinhibit themselves and disclose their private thoughts associated with a specific event anonymously to the researcher.

One possible explanation for the significant difference in avoidant thoughts but not intrusive thoughts is that the writing intervention forced the writers to think about one of the distressing experiences of their lives. The writing intervention helped the writers face, confront, and not avoid the hurtful places in their lives. But it may be that being reminded of these hurtful experiences was perceived as intrusive. Therefore, we did not find differences in intrusive thoughts concerning an experience written about and another experience not written about.

### Writing and General Distress

Consistent with our expectation (Hypothesis 3), the writing intervention did not directly translate into a reduction in general distress. While writers’ emotions and cognitions are about specific events, people, or objects, their general distress is less contextualized and less specific. The self-regulation perspective may help us understand this. People learn how to regulate their emotions by reflecting on successful experiences in emotion regulation. When children are young, a teacher or parent may guide them through the steps necessary to regulate their emotions successfully. Eventually, the child has enough experience, and they try what worked previously in new situations. This process involves a transfer of specific knowledge about what worked in one situation and applying that knowledge to another new situation.

We can expect that writers go through a similar self-regulation process when participating in a writing experiment. Although it is not the main function of these experiments, writing experiments teach participants how to regulate their emotions by writing. The participant is guided by following the writing instructions given in the experiment to regulate their emotions. Initially, there is no transfer of knowledge. The writer just follows the instructions. But if they repeat the instructions often enough and reflect on the experience, they may learn how to use these newfound skills with other difficult situations. This may be one reason why we did not observe differences in general distress as a direct outcome of the writing topic. Perhaps the skills learned in the writing experiment had not been repeated often enough to be applied to new situations and every situation generally. But it might be the case that we can see the early stages in this transference of knowledge. Consistent with our expectation (Hypothesis 4), the context specific negative emotions (upset and fear) and cognitions (avoidance and intrusion) were associated with general distress one week later. Additionally, feelings of being upset immediately after writing concerning the written-about event and the event not written about were differentially related to general distress one-week later. There is some evidence for an indirect connection: The writing intervention led to a reduction in being upset about a particular event. This decrease in being upset was associated with less general distress one week later.

### Implications, Limitations, and Future Directions

One implication of this study is that some of the cognitive and emotional benefits of writing about difficult life experiences are specific to the event written about. This means that targeting specific memories when writing or journaling may translate, at least in the short term, into benefits related to those memories. Moreover, the specific effects that one is most likely to experience include greater emotional strength, being less upset, and being less avoidant. These effects are likely to support writers’ self-concept, emotional stability, and coping with stressful events. Writing about difficult life experiences can be a therapeutic intervention carried out by practitioners or an easy self-help tool for individuals to improve social and emotional functioning. This study also outlines a methodology for studying the event-specific benefits of writing that should be replicated and extended.

A potential limitation of this study is the length, number, and spacing of sessions given to write. It could be argued that we did not ask the writers to write several times and for lengthy periods of time, and that consequently we did not find differences between the experimental and control groups comparable to those found in other studies. We take the argument. The length of writing time in previous writing studies has varied, with some asking participants to write as long as 30 min in one sitting (Greenberg, Wortman, & Stone, 1996) and others as short as 2 min (Burton & King, 2008). It has been suggested that writing about difficult life events is more effective if done on several occasions spaced over several days (e.g., Frattaroli, 2006; Páez et al., 1999). We agree. However, we focused on the benefits writers can reasonably expect to experience when writing briefly and intermittently. We felt that this brief writing closely mimicked the amount of time that we witness students and clients spend on writing. Therefore, when deciding how much the participants would write we favored external validity over internal validity, effectiveness over efficacy. We were also cautious about the degree to which we sensitized participants to the assessment procedure. Future research could ask writers to write over several occasions about the same event and then at the conclusion of the research have writers report their emotions and cognitions about two events. But it may be ill-advised to have multiple assessment points since this may sensitize the writers to the purpose of the study. This is also one reason why we chose random assignment to control for between-group differences rather than assessing all study variables before the experimental manipulation (Shadish, Cook, & Campbell, 2002).

Future research could examine whether these event-specific emotions and cognitions are associated with actions taken in the days immediately following the writing. It might be that writers find that they can channel their newfound strength and pride into their daily activities. Writers may also find that they are less avoidant of the people and places associated with their memories.

## Notes

For this article the following Supplementary Materials are available (Stapleton, Zhang & Berman, 2021):

- Writing instructions for participants.
